# Nd:YAG1064nm laser functions against *Sporothrix globosa* by inducing PANoptosis via the regulation of ZBP1-induced PANoptosome activation

**DOI:** 10.3389/fmicb.2025.1555338

**Published:** 2025-03-26

**Authors:** Tianyi Yan, Jinyan Nan, Rihua Jiang, Feng Chen, Jinran Li

**Affiliations:** ^1^Department of Dermatology, China-Japan Union Hospital of Jilin University, Changchun, China; ^2^Department of Dermatology, Second Hospital of Jilin University, Changchun, China

**Keywords:** Nd:YAG1064nm laser, PANoptosis, PANoptosome, pyroptosis, apoptosis, necroptosis, ZBP1, *Sporothrix globosa*

## Abstract

**Background:**

Due to the emergence of drug resistance in recent years, there is a need for new non-pharmacological treatment methods for sporotrichosis. Our previous study demonstrated that the Nd:YAG1064nm laser exhibited remarkable antifungal activity against *Sporothrix globosa*, but its exact mechanism remains unclear. This study aimed to detect PANoptosis regulatory protein ZBP1 expression in the skin lesions of patients with sporotrichosis, reveal the exact mechanism of Nd:YAG1064nm laser against sporotrichosis, and provide novel targets and methods for the diagnosis, assessment, and treatment of sporotrichosis.

**Methodology/principal findings:**

The ZBP1 level of 60 patients with sporotrichosis (≤3 months; *n* = 30 and >3 months; *n* = 30) and 30 HC were retrospectively reviewed using immunohistochemistry. The morphological changes, Hoechst/PI apoptosis and necroptosis preliminary exploration analysis, DNA fragmentation, calcium determination, and metacaspase activation were investigated *in vitro*. For the *in vivo* studies, mice were infected with *S. globosa* and then treated with a laser, and their footpad skin lesions and changes in the histology of tissue samples were compared. Changes in the levels of ZBP1, PANoptosome [RIPK1, RIPK3, Fas-associated death domain protein (FADD), CASP8], pyroptosis (CASP1, GSDMD), apoptosis (CASP3), and necroptosis (MLKL) related proteins were assessed using immunohistochemistry, whereas the levels of interleukin 17 (IL-17) and interferon gamma (IFN-γ) in peripheral blood were tested by enzyme-linked immunosorbent assay. ZBP1 expression was significantly increased in *S. globosa*-infected patients. Laser treatment effectively inhibited the growth of *S. globosa in vitro*, destroying its morphological structure, and maybe inducing apoptosis and necroptosis. Moreover, DNA fragmentation, calcium release into the cytoplasm, and metacaspase activation were observed. In addition, laser treatment demonstrated a clear therapeutic effect in animal models of sporotrichosis, which can lead to PANoptosis-related apoptosis, pyroptosis, and necroptosis. Immune response-related macrophages perceive nucleic acid level changes through ZBP1 to recognize *S. globosa* and induce PANoptosis by activating the PANoptosome (RIPK1/RIPK3/FADD/CASP8) complex with a Th1/Th17 cell response to combat sporotrichosis.

**Conclusion:**

Nd:YAG1064nm laser mediated PANoptosis resistance to sporotrichosis via ZBP1-PANoptosome-PANoptosis pathway.

## Introduction

*Sporothrix globosa* is the predominant etiologic agent of sporotrichosis worldwide, particularly in Northeast China (Moussa et al., 2017). In recent years, the global incidence of sporotrichosis, including many small-scale outbreaks and systemic infections, has been increasing (Rodrigues et al., 2022). Owing to the emergence of drug-resistant strains, it is difficult to cure them using clinical antifungal drug treatments (Liu et al., 2024). Therefore, exploring effective non-pharmacological treatment methods for sporotrichosis is important.

Laser therapy has the advantages of minimal adverse reactions, safety, effectiveness, and can be used as an antifungal treatment (Ribeiro et al., 2023; Yan et al., 2021a). Among the different types of lasers, the Nd:YAG1064nm laser has strong penetrability, precise control of the depth of skin penetration, minimal damage to surrounding tissues, a high mycological cure rate, and good safety (Bimbi, 2024). Recent studies have indicated that its thermal effect antagonizes fungal infections and initiates local immune responses (Baroni et al., 2018). At present, it is mainly used to treat various dermatological diseases, such as onychomycosis, candidiasis, and seborrheic dermatitis (Bimbi, 2024; Baroni et al., 2018; Liu et al., 2022). Our previous study showed that Nd:YAG1064nm laser had a significant therapeutic effect on animal models of sporotrichosis and could induce apoptosis, pyroptosis, and necrosis of sporotrichosis (Yan et al., 2021a). However, whether these mechanisms occur simultaneously or are interconnected remains unclear and requires further investigation.

PANoptosis is a recently discovered programmed cell death pathway that simultaneously initiates pyroptosis, apoptosis, and necroptosis. PANoptosis is regulated by PANoptosome complexes upon sensing pathogens, pathogen-associated molecular patterns (PAMPs), and damage-associated molecular patterns (DAMPs) (Christgen et al., 2020). Additionally, Z-DNA binding protein 1 (ZBP1), composed by Zα2 and RHIM domains, is a critical mediator of PANopotosis. Previous studies showed that various fungal infections could activate the Zα2 domain of ZBP1 by changes in nucleic acid levels, promoting the formation of PANoptosome complexes, and triggering PANoptosis (Samir et al., 2020; Zheng and Kanneganti, 2020). Banoth et al. (2020) demonstrated that PANoptosis-related apoptosis (CASP3/7/8), pyroptosis (CASP1 and GSDMD), and necroptosis (MLKL) proteins were significantly upregulated in macrophages infected with *Candida albicans* or *Aspergillus fumigatus*. Moreover, loss of PANoptosomes (RIPK1, RIPK3, CASP8, and FADD) inhibits macrophage death induced by *C. albicans* or *A. fumigatus* infection. The macrophages of ZBP1^−/−^ or ZBP1^ΔZα2/ΔZα^ mice are less susceptible to infection with *C. albicans* and *A. fumigatus*, indicating that both these agents can pass through the Zα2 domain of ZBP1 and promote the formation of PANoptosome (RIPK1, RIPK3, CASP8, and FADD) complexes, thereby activating PANoptosis by pyroptosis (NLRP3, ASC, CASP1/11, GSDMD), apoptosis (CASP3/7/8), and necroptosis (RIPK3/RIPK1, pMLKL). However, no study has found that *S. globosa* is associated with the ZBP1-PANoptosome-PANoptosis pathway after Nd:YAG1064nm laser treatment. Previous studies have shown that multiple types of lasers can induce DNA release, activate inflammasomes, and stimulate cytokines release (Yan et al., 2021a; Li et al., 2021). Our recent study showed that an Nd:YAG1064nm laser could destroy the morphological structure of *S. globosa*, blocking the cell cycle in the S phase and affecting DNA synthesis (Yan et al., 2021a). Therefore, we speculate that the Nd:YAG1064nm laser may activate the PANoptosis-related ZBP1 protein by causing changes in DNA levels in *S. globosa*. Indeed, the regulatory effects of the ZBP1-PANoptosome-PANoptosis pathway against sporotrichosis upon Nd:YAG1064nm laser treatment are worthy of further investigation.

This study aimed to investigate the inhibitory effect of Nd:YAG1064nm laser treatment on *S. globosa* and explore whether it occurs through the regulation of the ZBP1-PANoptosome-PANoptosis pathway.

## Materials and methods

### Patients and controls

Clinical data of 60 patients diagnosed with sporotrichosis at our hospital between January 2017 and December 2023 were retrospectively reviewed. The eligibility criteria were as follows: (1) adult patients and (2) an established sporotrichosis diagnosis based on histopathology and mycological culture (purulent secretion from the lesion). The exclusion criteria were as follows: (1) underage patients, (2) patients with diseases that affect ZBP1 expression, (3) patients with autoimmune diseases or those who have used immunosuppressants, and (4) patients who have used antifungal drugs or glucocorticoids within 3 months. For all sporotrichosis cases selected in this experiment, the clinical manifestations were fixed cutaneous and etiological agents were *S. globosa*. To analyze the effects of disease duration on host immunity, the included patients were divided into acute (≤3 months) and non-acute (> 3 months) groups based on onset or reactivation. Detailed clinical characteristics of the included patients with sporotrichosis are shown in Table 1. Thirty age- and sex-matched healthy controls (HCs) (aged 18–80 years; 14 males and 16 females) were selected for comparison with an average age of 51.39 ± 11.27 years. There were no statistically significant differences in gender and age between the acute group, non acute group and normal control group (*p* > 0.05).

**Table 1:** Clinical characteristics for 90 participants.

Parameters	Control group	Patient group	
		Acute group	Non-acute group
	(*n* = 30)	(*n* = 30)	(*n* = 30)
Age (years)	51.93 ± 11.27	48.33 ± 13.96	46.97 ± 13.06
Gender (m:f)	14:16	14:16	14:16
ZBP1 (AOD)	0.31 ± 0.05	0.54 ± 0.05***	0.45 ± 0.05***

Data are shown as mean ± SD; m, male; f, female. ^***^Mean compared to control group, *p* < 0.001.

The Institutional Review Boards of the China-Japan Union Hospital reviewed the study and approved the protocol. Informed consent was obtained from all patients and volunteers, Registration Number (20211130021).

### Immunohistochemistry

Immunohistochemistry (IHC) was performed to determine the levels of PANoptosis by detecting the expression of ZBP1 in paraffin sections from patients with sporotrichosis and HCs. Briefly, paraffin-embedded sections were dewaxed and gradually rehydrated before immersion in an EDTA solution (pH 9.0). After the paraffin-embedded sections were heated at 120°C for 150 s, the sections was slowly cooled to room temperature. After fixation, the sections were blocked with immunostaining blocking solution and incubated with primary antibody at 4°C overnight. The sections were then treated with a rabbit IGF2BP1 (ZBP1) polyclonal antibody (DF6436, 1:1600; Affinity Biosciences, Cincinnati, OH, United States). After washing with PBS, the sections were incubated with horseradish peroxidase (HRP)-conjugated secondary antibodies for 20 min at 37°C, visualized with diaminobenzidine, and counterstained with hematoxylin, followed by treatment with hydrochloric acid ethanol for differentiation, phosphate buffer solution for returning to blue, ethanol gradient for dehydration, xylene for transparency, and neutral gum for sealing.

### *Sporothrix globosa* preparation and culture conditions

*S. globosa* isolated from strains preserved in our laboratory was used. Molecular identification and mycelial-to-yeast phase conversion cultures were used to identify *S. globosa* strains (Yan et al., 2020). All isolates were grown on Sabouraud dextrose agar for 7 days. The fungal colonies were then transferred to brain-heart infusion broth and incubated in a rotary shaker (150 rpm) at 37°C for 7 days. After centrifugation, *S. globosa* cells were resuspended in PBS and diluted to 1 × 10^8^ cells/mL. Two microliter aliquots (per drop) of each dilution were inoculated on brain-heart infusion broth agar plates and incubated for 7 days before laser irradiation.

### *In vitro* Nd:YAG1064nm laser irradiation

*S. globosa* was inoculated into a brain heart solid culture dish (six drops per dish, including three drops in the laser irradiation group and three drops in the control group) on a clean bench. After 7 days, 60 colonies were randomly and blindly allocated to the control (*n* = 30) and study (*n* = 30) groups. The colonies from the control group were not treated. Colonies of the study group were treated with an Nd:YAG1064nm laser (Jilin Provincial King Laser Technology Co., Ltd. Changchun, China) using the following settings (according to our pre-experiment of the minimum energy density that inhibits the growth of *S. globosa* by more than 50%; the actual energy density absorbed by *S. globosa* cells was calculated as follows: actual energy density = *b*/*πr*^2^, where *b* is the actual energy absorbed by *S. globosa* cells and *r* is the spot semidiameter of the laser beam); power of 160 J/cm^2^, the spot diameter of the laser beam was 4 mm, with the laser emitting 30-ms pulses at a 1-Hz pulse/frequency, and the actual energy density received by *S. globosa* cells was 114 J/cm^2^, which is consistent with commonly used clinical doses. During laser irradiation, the laser head was positioned 5 mm away from the plate. The laser beam irradiated the entire colony and moved in a spiral shape from the periphery to the center. Each colony was irradiated with 8 to 10 light spots. After the entire colony was irradiated, a 2-min pause was applied. The treatment and pause were repeated three times before the follow-up study.

### Observation of *Sporothrix globosa* morphology after laser treatment

To visualize the morphology of *S. globosa* after laser treatment, the cells were examined as described below. The strains in the laser and control groups were cultured in 24-well tissue culture plates with cell slides for 4 h. After discarding the supernatant and washing each well with PBS three times, both cell groups were fixed with 2.5% glutaraldehyde for 2 h. After fixation, the disks were rinsed three times with PBS and dehydrated in an ethanol series (30, 50, 70, 80, and 90% for 7 min each, and 100% for 10 min). The disks were dried at the conventional critical point and vacuum-sprayed with a gold coating. Finally, the morphology of *S. globosa* was observed using a scanning electron microscope (SEM).

### Preliminary exploration of cell death pathways after laser treatment

Hoechst 33342-propidium iodide (PI) double-staining detection kit (Beyotime Institute of Biotechnology, Jiangsu, China) were used to preliminary explore whether apoptosis and necroptosis were occurred after laser treatment. After laser treatment for 4 h, the strains of the laser and control groups were centrifuged at 3,000 rpm for 5 min and then resuspended at 800 μL staining buffer. The strain solution was rinsed twice with PBS and then diluted to 1 × 10^6^ CFU/mL with a hemocytometer. Five microliter of Hoechst 33342 and 5 μL of PI were incubated into the solution and mixed according to the manufacturer’s instructions. The solution was washed once with PBS in an ice bath for 30 min. The *S. globosa* cells were observed under a laser-scanning confocal microscope (Zeiss LSM 780, Germany).

### Analysis of DNA fragmentation

DNA fragmentation was detected using a One Step TUNEL Apoptosis Assay Kit (Beyotime Institute of Biotechnology, Shanghai, China). After laser treatment for 4 h, *S. globosa* cells in the laser and control groups (1 × 10^6^ CFU/mL) were washed twice with PBS and fixed for 30 min. *S. globosa* was resuspended in a strong immunostaining penetrant solution and incubated at room temperature for 5 min. A TUNEL detection solution was prepared and mixed thoroughly. The *S. globosa* was washed with PBS twice before adding 50 μL TUNEL detection solution and incubating at 37°C in the dark for 60 min. After washing with PBS, cells were analyzed using a flow cytometer at excitation and emission wavelengths of 450 and 550 nm, respectively.

### Cytosolic calcium determination

The effects of laser irradiation on the cytosolic calcium levels were tested using a Fluo-4 Calcium Assay Kit (Beyotime Institute of Biotechnology, China). Briefly, the treated *S. globosa* in the laser and control groups (1 × 10^6^ CFU/mL) were centrifuged and washed twice with PBS. After precipitation, 1 mL Fluo-4 staining solution was added, and the cells were resuspended. After incubation at 30°C for 30 min in the dark, the *S. globosa* was washed once and centrifuged. A total of 0.5 mL of detection buffer was added to each sample to resuspend the *S. globosa* and incubated at 30°C for an additional 20 min. The fluorescence intensity of Fluo-4 AM (excitation = 490 nm, emission = 525 nm) was measured immediately using a flow cytometer.

### Metacaspase activation assay

Metacaspase activity was measured using a CaspACE FITC-VAD-FMK in-situ marker (Promega, Madison, WI, United States). The strains from the laser and control groups were centrifuged at 3,000 rpm for 5 min and diluted to 1 × 10^6^ CFU/mL. *S. globos*a was washed once with PBS before staining with 10 μM CaspACE FITC-VAD-FMK. After 20 min of incubation at 37°C, *S. globosa* was washed twice with PBS and the fluorescence absorption was detected using laser scanning confocal microscope (Zeiss LSM 780, Germany). Fluorescence absorption was measured using a microplate fluorescence reader at excitation and emission wavelengths of 494 and 530 nm, respectively.

### Mice

A total of 39 female BALB/c mice (age, 8–10 weeks; body weight, 16–20 g) were purchased from Changchun Yisi Experimental Animal Technology Co., Ltd., Jilin, China. The animal protocol was approved by the Institutional Ethics Committee for Animal Use in Research, and animal care followed the guidelines of Jilin University. The mice were housed in groups of six per cage. The mice were maintained under a 12:12-h light:dark cycle with free access to food and water. The mice were allocated to the laser (*n* = 12), infection (*n* = 15), and healthy control (HC) groups (*n* = 12).

### Inoculation and antifungal treatments

After routine sterilization, 0.05 mL of an *S. globosa* suspension (1 × 10^8^ CFU/mL) was injected into the footpads of mice in the laser and untreated infection groups. The skin conditions were monitored daily. On day 10, histopathological analysis (*n* = 3) confirmed that the mouse models were successfully established. In the laser group, the injected feet were subjected to Nd:YAG1064nm laser irradiation at room temperature with an irradiation energy density, 100 J/cm^2^; spot diameter, 4 mm; pulse duration, 30 ms; pulse frequency, 1 Hz. Laser treatment was administered on days 10 and 18.

On days 18 and 26, six mice from each group were euthanized, and blood and foot tissue samples were taken to perform skin histology, IHC, and serum cytokine levels.

### Skin histology

Fungal inoculation sites on the mouse skin were isolated and fixed with 10% buffered formalin for at least 24 h. The samples were then dehydrated, embedded in paraffin, and sliced into 5-mm sections. To analyze skin inflammation, sections were stained with hematoxylin and eosin. The ImageJ software was used to quantify the percentage area of inflammatory cells on days 18 and 26.

### IHC

IHC was used to determine the levels of PANoptosis by detecting the expression of ZBP1, PANoptosomes (RIPK1, RIPK3, CASP8, and FADD), pyroptosis (NLRP3 and GSDMD), apoptosis (CASP3), and necroptosis (MLKL) in paraffin sections of mouse footpad tissues. Briefly, paraffin-embedded sections were dewaxed and gradually rehydrated before immersion in an EDTA solution (pH 9.0). After the paraffin-embedded sections were heated at 120°C for 150 s, the sections was slowly cooled to room temperature. After fixation, the sections were blocked with immunostaining blocking solution and incubated with primary antibody at 4°C overnight. The sections were treated with the following primary antibodies: rabbit IGF2BP1 (ZBP1) polyclonal antibody (DF6436, 1:2,000), rabbit CASP8 polyclonal antibody (AF6442, 1:2,000), rabbit Phospho-FADD (Ser194) polyclonal antibody (DF2996, 1:2,000), rabbit GSDMD polyclonal antibody (AF4012, 1:2,000), and rabbit MLKL polyclonal antibody (DF7412, 1:2,000) (all antibodies were sourced from Affinity Biosciences, Cincinnati, OH, United States). Rabbit CASP1 monoclonal antibody (AF1681, 1:400) and CASP3 rabbit polyclonal antibody (AF0081, 1:100) were purchased from Beyotime Institute of Biotechnology (Jiangsu, China). Rabbit RIPK3 (bs-3551R, 1:2,000) and rabbit RIPK1 polyclonal antibodies (bs-5805R, 1:2,000) were sourced from Bioss Biological Technology (Wuhan, China). After washing with PBS, the sections were incubated with horseradish peroxidase (HRP)-conjugated secondary antibodies for 20 min at 37°C, visualized with diaminobenzidine, followed by counterstaining with hematoxylin, hydrochloric acid ethanol differentiation, phosphate buffer solution back blue, gradient ethanol dehydration, xylene transparent, and neutral gum seal.

### Enzyme-linked immunosorbent assay

Peripheral blood mononuclear cells were separated from whole blood samples drawn from euthanized BALB/c mice using Ficoll density gradient centrifugation. The serum levels of IL-17 and IFN-*γ* were measured using enzyme-linked immunosorbent assay (ELISA) kits (Mlbio, Shanghai, China) according to the manufacturer’s instructions. Absorbance was measured at 450 nm using a 96-well plate reader.

### Statistical analysis

All statistical analyses were performed using SPSS version 24 (SPSS, Armonk, NY, United States). The normal distribution and variance homogeneity were measured then data were summarized as the median values and ranges for non-normally distributed data or the mean ± standard deviations for normally distributed data. Quantitative analysis of IHC was performed by assessing the average optical density (AOD) using ImageJ. The Mann–Whitney *U* test and Student’s *t*-test were performed to evaluate between-group differences. Pearson’s correlation analysis and Spearman rank correlation analysis were used for analyzing associations between the variables. Statistical significance was set at *p* < 0.05.

## Results

### *Sporothrix globosa* infection triggers the activation of ZBP1

**Figure 1:**
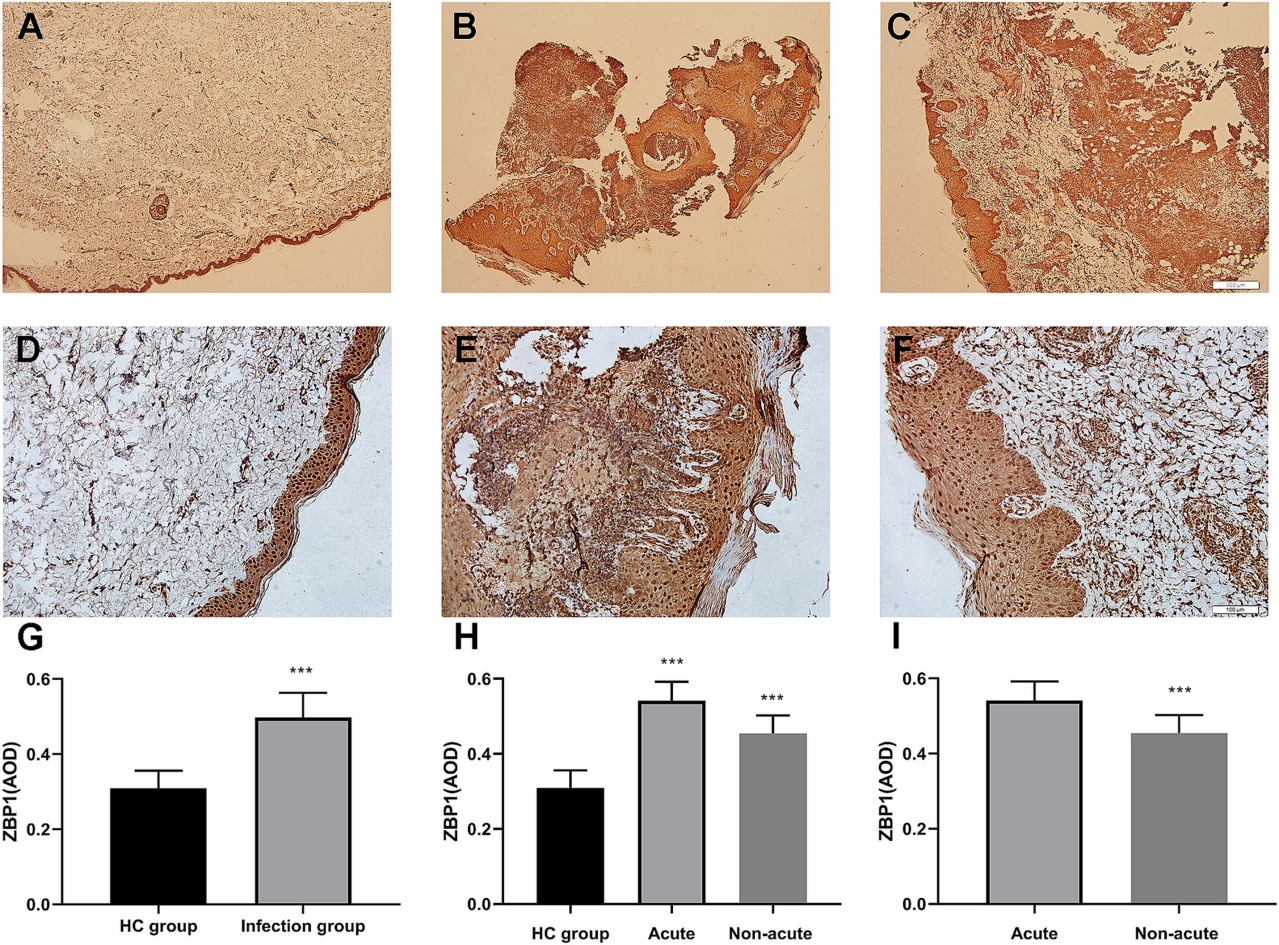
Immunohistochemical analysis demonstrated that ZBP1 was more highly expressed in infection tissues compared with control tissues. **(A,D,G)** Control group. **(B,E,H)** Acute infection group. **(C,F,I)** Non-acute infection group. Red agent was added to distinguish positive staining from skin pigmentation, with a positive result considered as red staining. Magnification: **(A–C)** ×40, **(D–F)** ×200. ZBP1, Z-DNA binding protein 1. ^***^p < 0.001 indicate statistical significance.

The characteristics of the study participants are summarized in Table 1. The expression of ZBP1 in the skin lesions of patients with sporotrichosis and HCs was detected using IHC using the average optical density (AOD) to evaluate positivity (Table 1 and Figure 1). Compared with the HC group (0.31 ± 0.05), we found that ZBP1 levels were upregulated in the infection group (0.50 ± 0.07; *p* < 0.001). The expression level of ZBP1 was unrelated to sex and age (*p* > 0.05) but was related to the course of the disease. The expression level of ZBP1 in the acute group (0.54 ± 0.05) was significantly higher than that in the non-acute group (0.45 ± 0.05; *p* < 0.001). These data suggest that the PANopotosis-related protein, ZBP1, is activated after *S. globosa* infection.

### *Sporothrix globosa* morphological changes after laser irradiation

**Figure 2:**
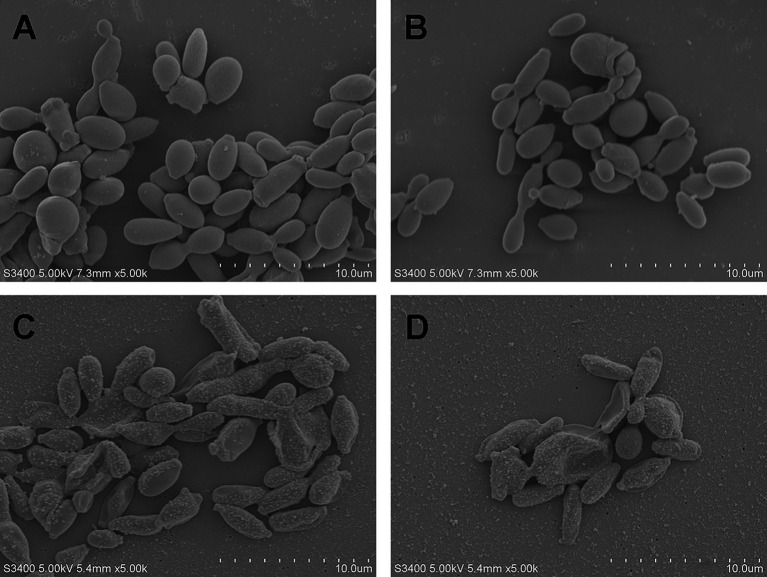
Morphological changes of *S. globosa* after laser treatment. **(A,B)** The normal *S. globosa* showed regular oval shape, complete and smooth surface, clear borders with no wrinkles. **(C,D)** After Nd:YAG1064nm laser treatment, *S. globosa* surface showed many granular or vesicle-like structures, some areas show irregular shapes.

SEM was performed to investigate the effect of the Nd:YAG1064nm laser on the morphology of *S. globosa*. *S. globosa* showed normal morphology in the control group, with a regular oval shape, complete and smooth surface, and clear borders with no wrinkles (Figures 2A,B). In the Nd:YAG1064nm laser irradiation group, significant morphological changes in *S. globosa* were observed, with many granular or vesicle-like structures on the surface and some areas showing irregular shapes such as depressions, holes, looseness, and fragmentation (Figures 2C,D).

### *Sporothrix globosa* cell death pathways after laser irradiation

**Figure 3:**
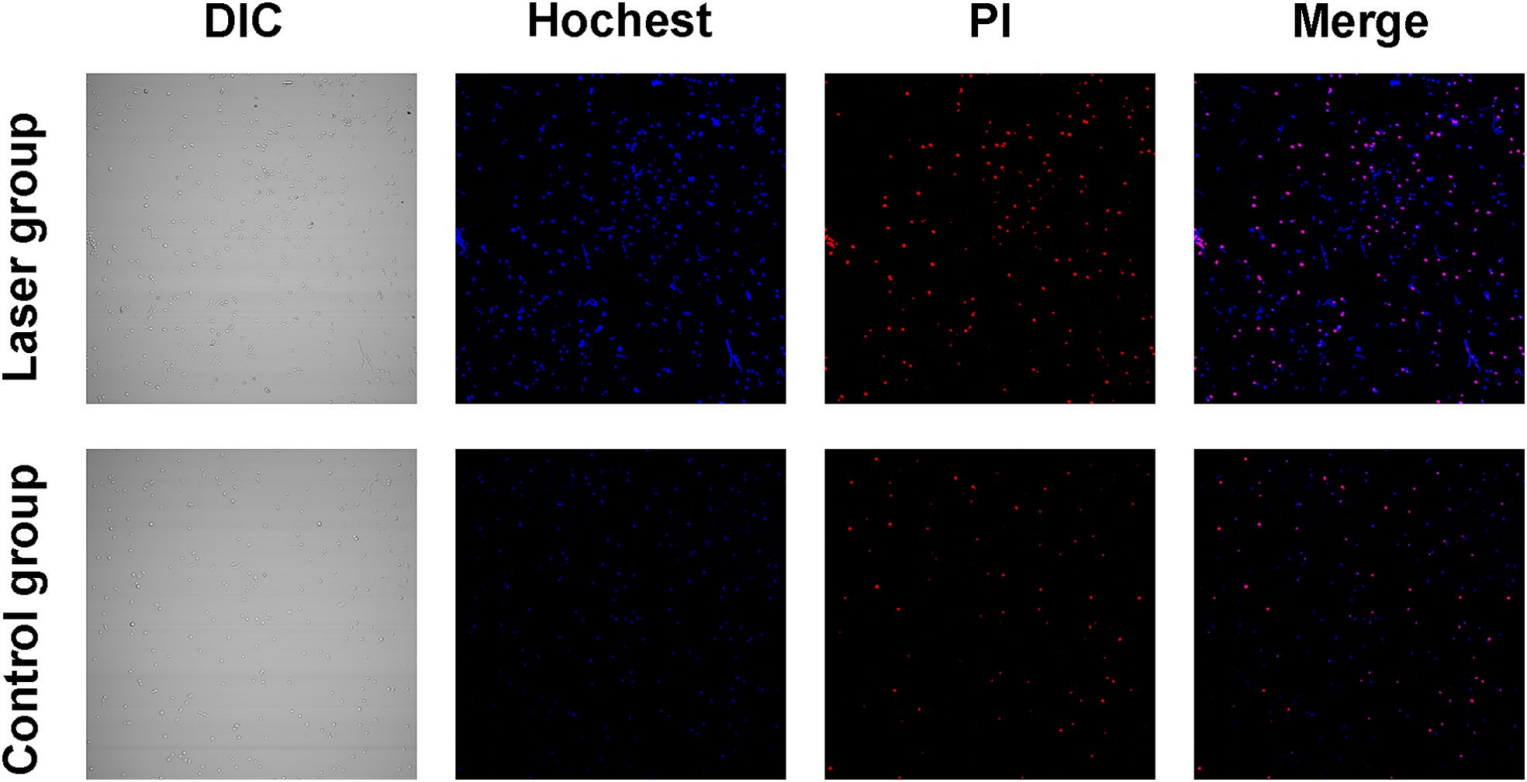
Apoptosis and necroptosis effect of *S. globosa* after laser treatment. Confocal laser scanning microscopy was used to evaluate the effects of Nd:YAG1064nm laser treatment on apoptosis and necroptosis in *S. globosa* of the laser and control groups as observed by Hoechst/PI double staining. DIC, differential inference contrast.

To preliminary explore the effect of Nd:YAG1064nm laser on cell death pathways, Hoechst/PI double staining was performed. Apoptotic *S. globosa* cells showed weak red and strong blue fluorescence. Necrotic *S. globosa* cells showed strong red and blue fluorescence. Only weak blue and red fluorescence was observed in the control group, indicating that *S. globosa* was in a normal state. However, strong blue and red fluorescence (Hoechst^+^/PI^+^) was observed in the Nd:YAG1064nm laser irradiation group (Figure 3), suggesting that Nd:YAG1064nm laser treatment maybe significantly promoted apoptosis and necroptosis in *S. globosa*.

### Laser triggers DNA fragmentation and elevates cytosolic Ca^2+^ levels in *Sporothrix globosa*

**Figure 4:**
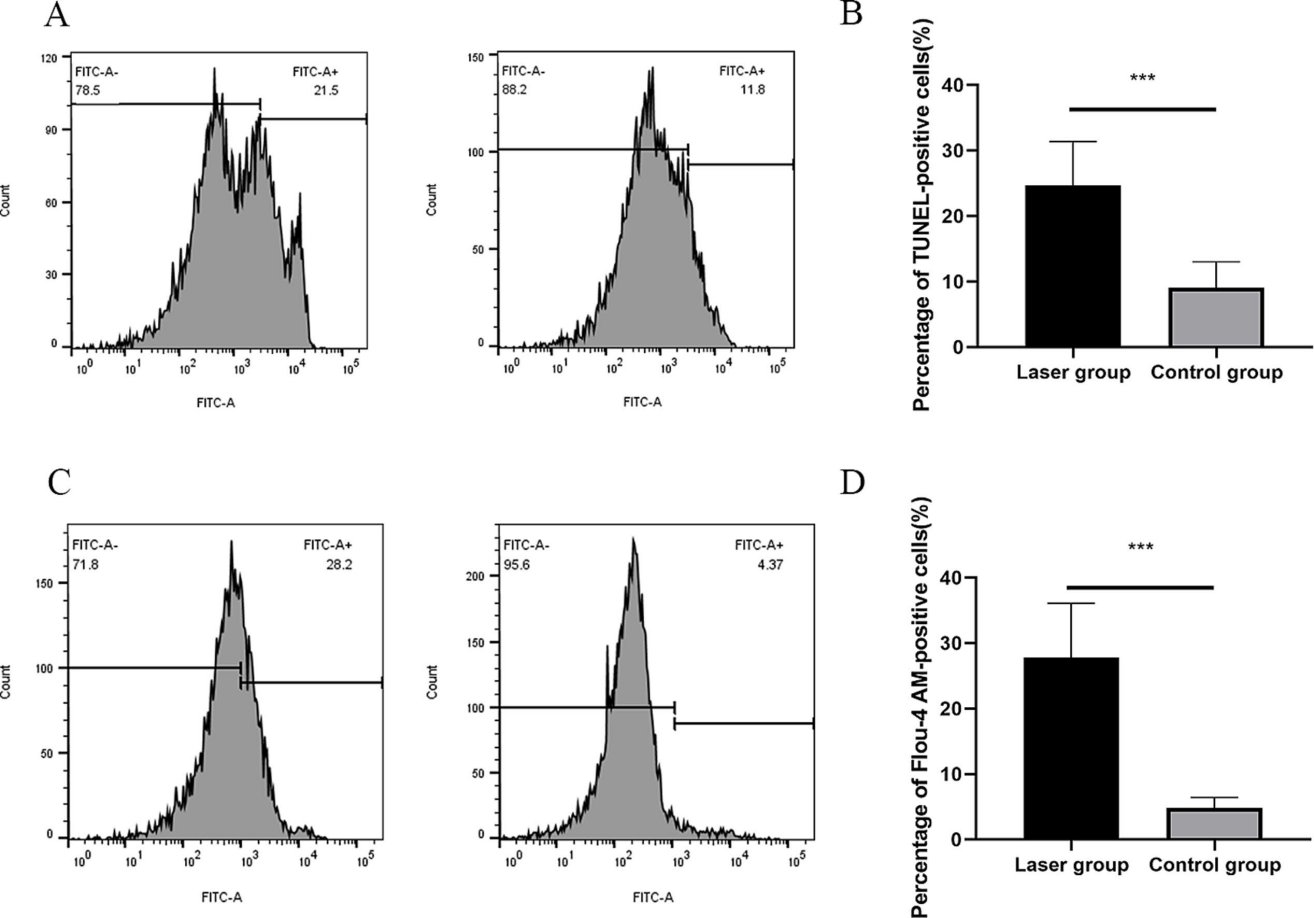
DNA fragmentation and cytosolic Ca^2+^ Levels of *S. globosa* after Nd:YAG1064nm laser treatment. **(A,B)** Flow cytometer was used to evaluate the effects of laser treatment on DNA Fragmentation in *S. globosa* of the laser and control groups as observed by DAPI staining. **(C,D)** Flow cytometer were used to detect the effects of Nd:YAG1064nm laser treatment on cytosolic Ca^2+^ levels in *S. globosa* of the laser and control groups as observed by staining with Fluo-4 AM. ^***^p < 0.001 indicates statistical significance.

ZBP1 is the key innate immune sensor for endogenous nucleic acid ligands, which regulate PANoptosis by sensing changes in nucleic acid levels; therefore, DNA fragmentation levels were detected (Zheng and Kanneganti, 2020). The exposed 3′-OH is catalyzed by Terminal Deoxynucleotide Transferase after DNA breaks. The addition of fluorescein dUTP labeled with the green fluorescent probe FITC can detect these changes (Jia et al., 2019). An enhanced fluorescence intensity was observed with an increase after Nd:YAG1064nm laser treatment (9.10 ± 3.91% vs. 24.67 ± 6.71%; *p* < 0.001) (Figures 4A,B), implying laser caused DNA fragmentation.

Previous study showed that increased cytosolic Ca^2+^ triggers mitochondrial permeabilization and releases pro-apoptotic factors that initiate programmed cell death (Jia et al., 2019). In this study, Fluo-4 AM was used to measure the cytosolic Ca^2+^ levels. Data showed that the fluorescence intensities of Fluo-4 AM was observed to significantly increase in *S. globosa* treated with Nd:YAG1064nm laser (4.81 ± 1.61% vs. 27.80 ± 8.28%; *p* < 0.001) (Figures 4C,D), suggesting that Nd:YAG1064nm triggered Ca^2+^ accumulation in the cytosol.

### *Sporothrix globosa* caspase activity after laser irradiation

**Figure 5:**
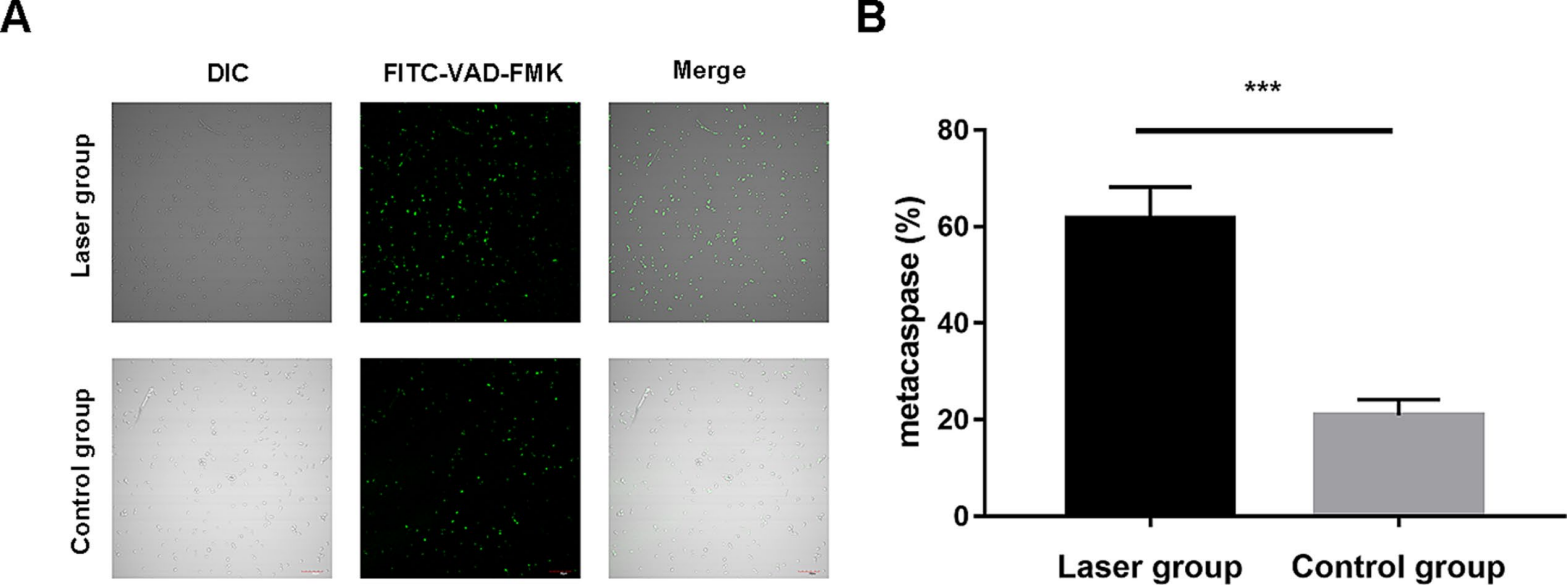
Metacaspase activation of *S. globosa* after Nd:YAG1064nm laser treatment. **(A)** Confocal laser scanning microscopy and **(B)** microplate fluorescence reader were used to detect the effects of Nd:YAG1064nm laser treatment on metacaspase activation in *S. globosa* of the laser and control groups as observed by staining with CaspACE FITC-VAD-FMK. DIC, differential inference contrast. ^***^p < 0.001 indicate statistical significance.

Meta-caspases are caspase orthologs that play important regulatory roles in fungal apoptosis. Only a small amount of fluorescence is observed in the control group. However, in the laser treatment group, most cells showed bright green fluorescence with the intensities of 20.89 ± 3.30% vs. 61.63 ± 6.54%; *p* < 0.001 using the microplate fluorescence reader, indicating that the Nd:YAG1064nm laser irradiation activated the caspase-dependent cell apoptosis pathway in *S. globosa* (Figures 5A,B).

### Size of laser-irradiated mouse footpads after *Sporothrix globosa* infection

**Figure 6:**
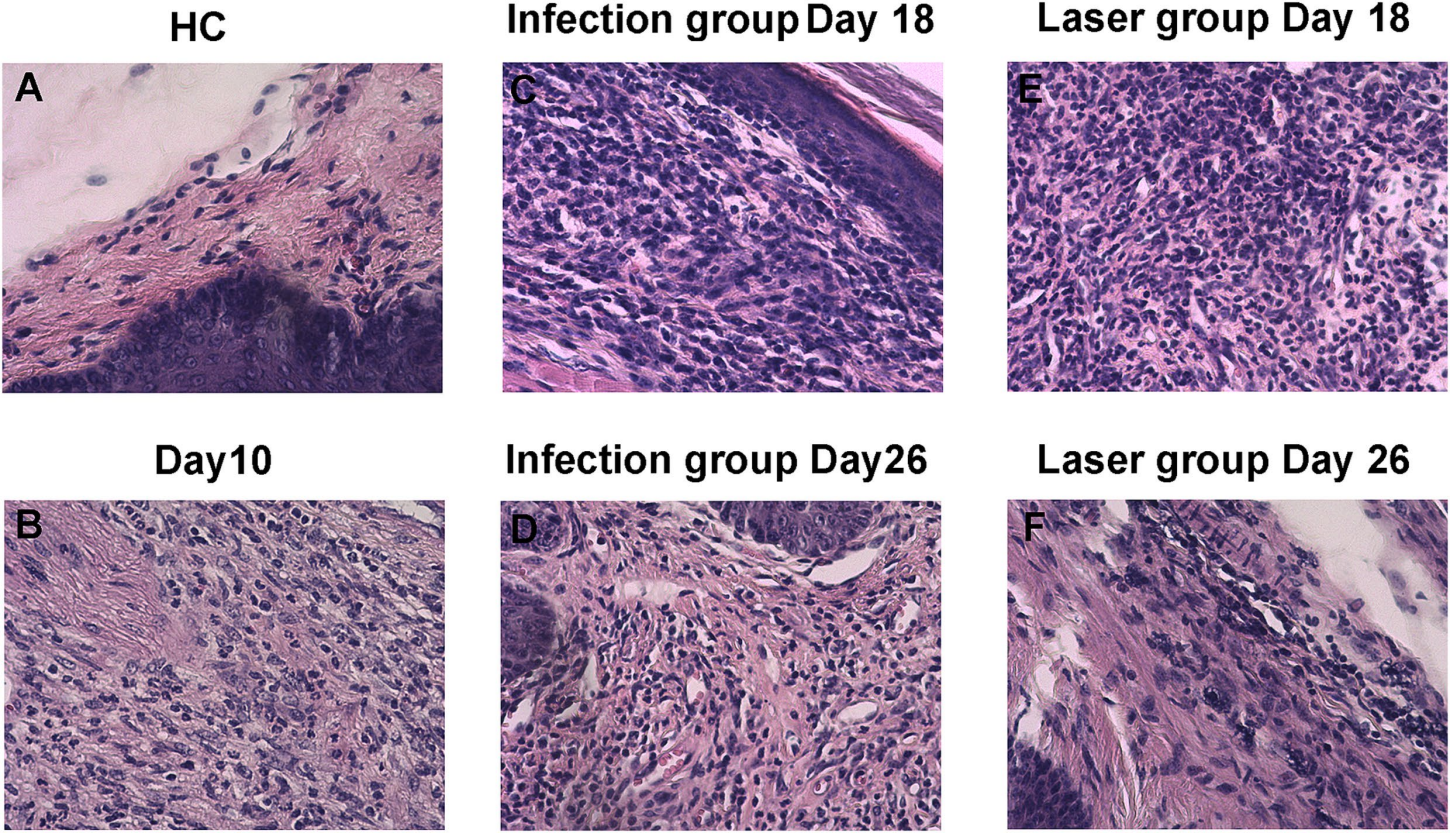
Histopathology of skin tissues of *S. globosa*-infected mice on day 18 and 26 after different treatments. **(A)** Healthy control (HC) group. **(B)** Study group on day 10. **(C–F)** Histopathological changes of skin tissues of the different treatment groups on day 18 and 26.

**Figure 7:**
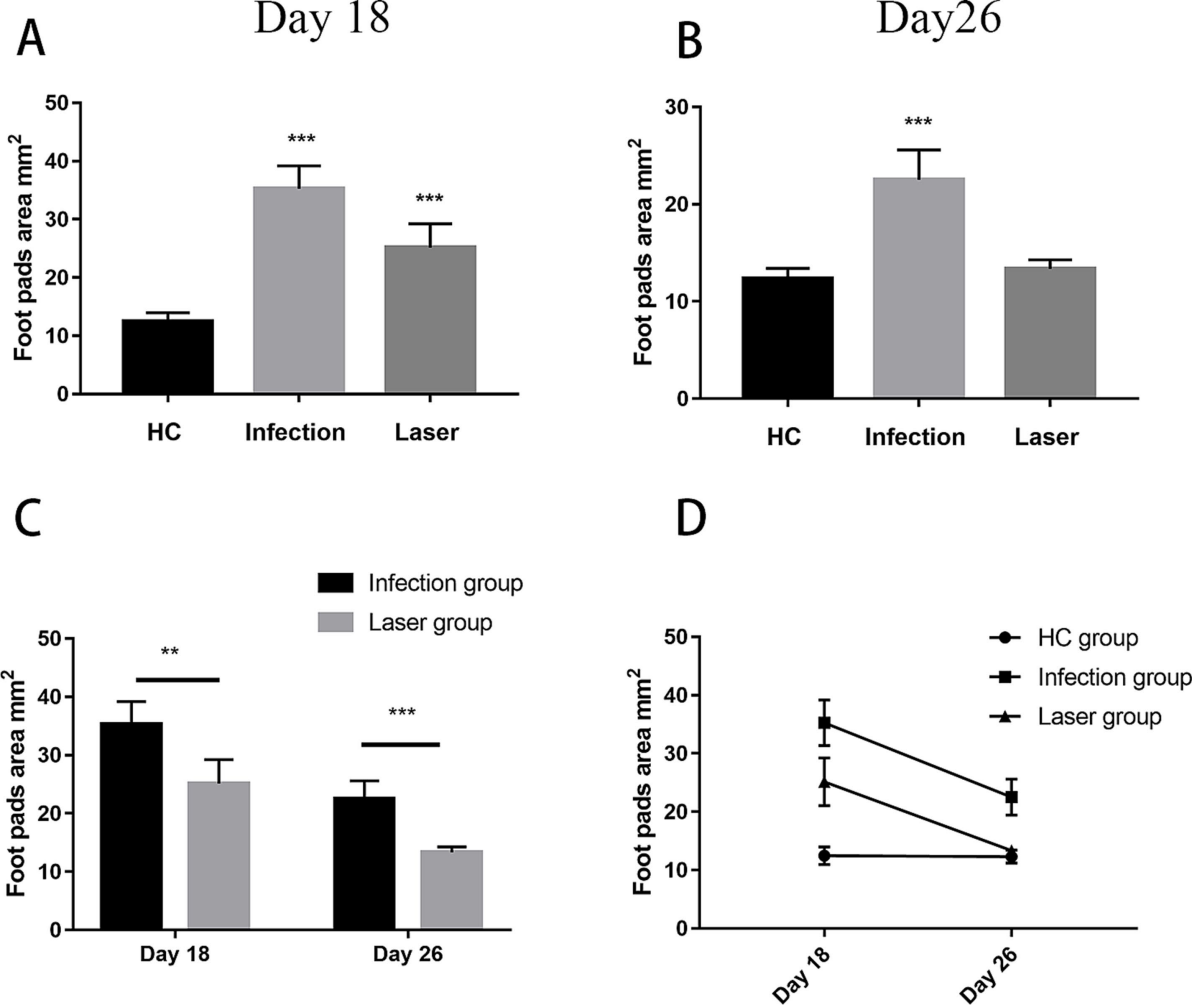
The footpad sizes of mice after different treatments. **(A,B)** Compared with the HC group, the footpads’ size after different treatments on day18 and 26. **(C)** Compared with the Infection group, the footpads’ size in laser group from day18 to 26. **(D)** Analysis on the trend of the skin lesion area of all groups mice followed up time from day18 to 26. ^**^p < 0.01 and ^***^p < 0.001 indicate statistical significance.

In the study group, the footpads of the mice began to become red and swollen on day 2 after *S. globosa* inoculation. Subsequently, the swelling gradually increased with ulceration, suppuration, and significant inflammatory lumps. On day 10, the *S. globosa* infection model was successfully established as confirmed by three histopathological tests (Figure 6B). Next, the mice were divided into an infection group (*n* = 12) and a laser treatment group (*n* = 12). We found that on day 18, the footpad sizes of mice were significantly larger in the infection (35.27 ± 3.93 mm^2^; *p* < 0.001) and laser (25.15 ± 4.07 mm^2^; *p* < 0.001) group than that in the HC group (12.49 ± 1.51 mm^2^) (Table 2 and Figure 7A). On day 26, the footpad sizes of the laser group (13.36 ± 0.92 mm^2^; *p* = 0.108) had returned to nearly normal (12.33 ± 1.09 mm^2^), a faster recovery was observed in the infection group (22.51 ± 3.08 mm^2^; *p* < 0.001) (Figure 7B). From days 18 to 26, a significant decrease in the size of mouse footpads was observed in the laser group compared to the infection group (Figures 7C,D).

**Table 2:** The mice footpad sizes.

Parameters	Study group	Day 18	Day 26
		(*n* = 6)	(*n* = 6)
Mean footpad area ± SD (mm^2^)	HC group	12.49 ± 1.51	12.33 ± 1.09
	Infection group	35.27 ± 3.93^***^	22.51 ± 3.08^***^
	Laser group	25.15 ± 4.07^***^	13.36 ± 0.92

Normally distributed data are shown as the mean ± SD. ^***^Mean compared to HC, *p* < 0.001.

### Histopathology of laser-irradiated mouse after *Sporothrix globosa* infection

The skin histopathology of the HC mice is shown in Figure 6A. After 10 days of infection, three mice were randomly selected for histopathological analysis. We observed a large number of epithelioid cells and lymphocytes as well as granulomatous changes, indicating that the mouse model was successfully constructed (Figure 6B). The histopathological changes in the mouse footpads of the different groups were further analyzed on days 18 and 26 (Figures 6C–F). On day 18, suppurative granulomas with numerous neutrophils and lymphocytes were observed in the infection and laser-treated groups (Figures 6C,E). On day 26, although inflammation remained in the infection group, the mouse histopathology in the laser group almost returned to normal, and the pus-like inflammatory foci almost disappeared (Figures 6D,F). We demonstrated that the Nd:YAG1064nm laser promoted mouse footpad recovery, which may be used as an alternative for clinical *S. globosa* treatment.

### ZBP1 expression of laser-irradiated mouse after *Sporothrix globosa* infection

**Figure 8:**
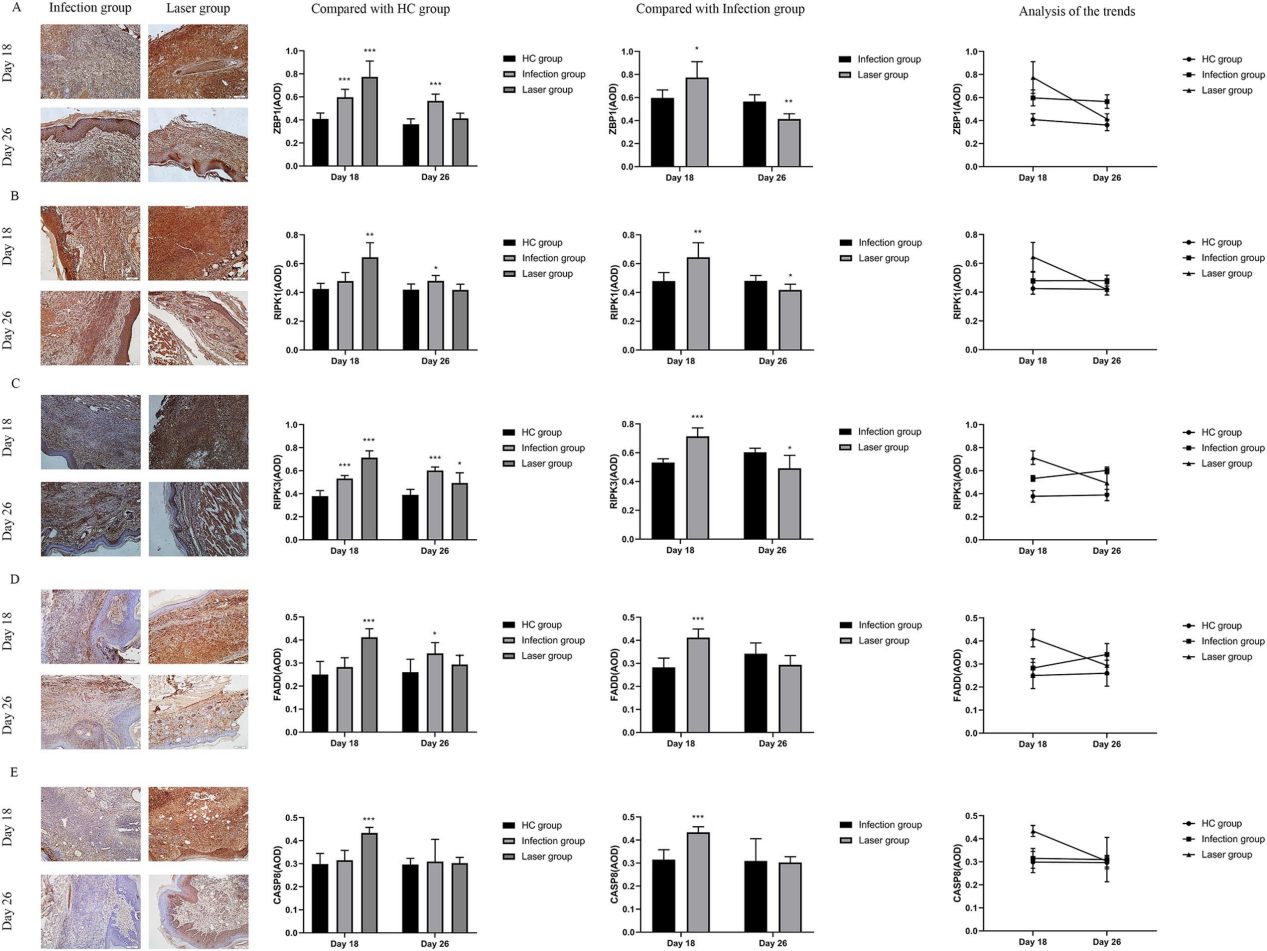
Immunohistochemical analysis of ZBP1 and PANoptosome expression levels in the tissues of *S. globosa*-infected mice after different treatments on day 18 and 26. **(A)** ZBP1. **(B)** RIPK1. **(C)** RIPK1. **(D)** FADD. **(E)** CASP8. Red agent was added to distinguish positive staining from skin pigmentation, with a positive result considered as red staining. Magnification: ×200. ZBP1, Z-DNA binding protein 1; RIPK1, recombinant receptor interacting serine threonine kinase 1; RIPK3, recombinant receptor interacting serine threonine kinase 3; FADD, Fas-associating protein with a novel death domain; CASP8, caspase-8; AOD, average optical density. ^*^p < 0.05, ^**^p < 0.01, and ^***^p < 0.001 indicate statistical significance.

ZBP1 is a nucleic acid sensing protein, including Zα2 domain and RHIM domain. Previous studies have shown that ZBP1, a central regulator of PANoptosis, can regulate cell apoptosis, pyroptosis, and programmed necrosis through the PANoptosome, thereby helping the host resist infection (Samir et al., 2020; Zheng and Kanneganti, 2020). In the *in vivo* study, we further measured the expression ZBP1 in mice footpad tissues from the different groups. Compared with the HC group (0.41 ± 0.05), our data showed that ZBP1 levels were upregulated in the laser (0.77 ± 0.14; *p* < 0.001) and infection (0.60 ± 0.07; *p* < 0.001) groups on day 18. Furthermore, the ZBP1 levels in the laser group were significantly higher than those in the infection group (*p* = 0.018), indicating that ZBP1 was significantly activated by laser treatment *in vivo* (Figure 8A). On day 26, the ZBP1 levels of the infection group (0.57 ± 0.06; *p* < 0.001) remained higher than that of the HC group (0.36 ± 0.05), whereas the levels returned to almost normal in the laser group (0.41 ± 0.05; *p* = 0.082) (Figure 8A), indicating that the therapeutic effect of laser on mice skin tissues may be related with the ZBP1expression.

### PANoptosome expression of laser-irradiated mouse after *Sporothrix globosa* infection

The PANoptosome comprises RIPK1, RIPK3, FADD, and CASP8. Previous studies found that both *C. albicans* and *A. fumigatus* could promot PANoptosome and activate PANoptosis through the Zα2 domain of ZBP1 after infection (Banoth et al., 2020). *S. globosa* is most similar to *C. albicans*. In this study, we investigated whether PANoptosomes were activated by ZBP1 in laser-irradiated mice after *S. globosa* infection. Compared with the HC group, our data showed that RIPK1 (0.64 ± 0.10, 0.42 ± 0.04; *p* = 0.001), RIPK3 (0.71 ± 0.06, 0.38 ± 0.05; *p* < 0.001), FADD (0.41 ± 0.04, 0.25 ± 0.06; *p* < 0.001) and CASP8 (0.43 ± 0.02, 0.30 ± 0.05; *p* < 0.001) levels were all upregulated in the laser group on day 18 (Figures 8B–E). Furthermore, RIPK1 (0.64 ± 0.10, 0.48 ± 0.06; *p* = 0.006), RIPK3 (0.71 ± 0.06, 0.53 ± 0.03; *p* < 0.001), FADD (0.41 ± 0.04, 0.28 ± 0.04; *p* < 0.001) and CASP8 (0.43 ± 0.02, 0.32 ± 0.04; *p* < 0.001) levels in the laser group was significantly higher than that in the infection group, indicating that the PANoptosome was activated after *S. globosa* infection, whereas laser treatment further strengthened its expression *in vivo* (Figures 8B–E). On day 26, the RIPK1 (0.48 ± 0.04, 0.42 ± 0.04; *p* = 0.021), RIPK3 (0.60 ± 0.03, 0.39 ± 0.05; *p* < 0.001), and FADD (0.34 ± 0.05, 0.26 ± 0.06; *p* = 0.021) levels of the infection group was still higher than that of the HC group, while the RIPK1 (0.42 ± 0.04, 0.42 ± 0.04; *p* = 0.966), FADD (0.29 ± 0.04, 0.26 ± 0.06; *p* = 0.261), and CASP8 (0.30 ± 0.03, 0.30 ± 0.03; *p* = 0.707) level returned to almost normal in the laser group (Figures 8B–E).

**Figure 9:**
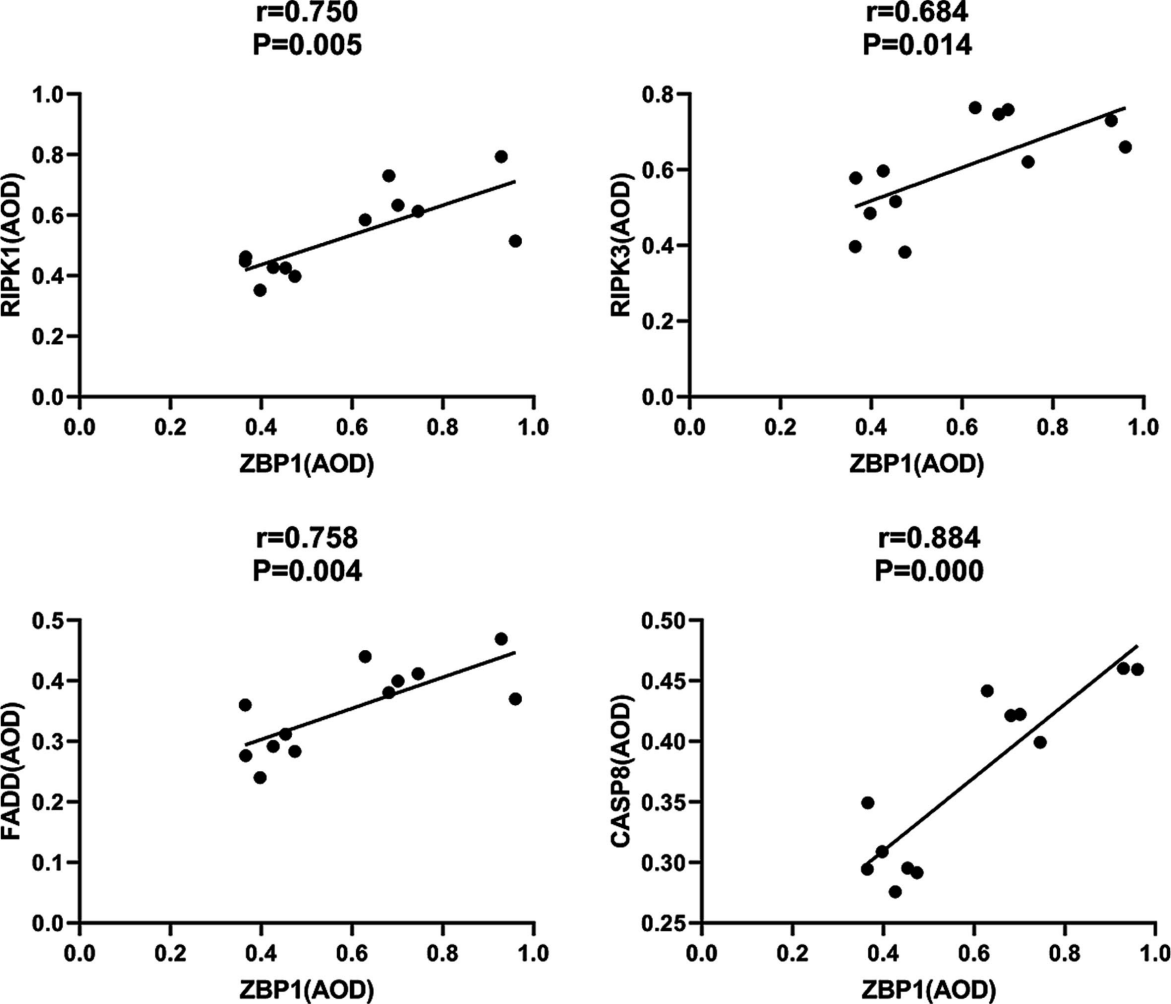
Correlations between ZBP1 and the composition of PANoptosome (RIPK1, RIPK3, FADD, and CASP8) levels in *S. globosa*-infected mice after laser irradiation. Positive correlations between ZBP1 and RIPK1, RIPK3, FADD, CASP8 levels after laser irradiation by IHC. AOD, average optical density.

In addition, we found that the PANoptosome of RIPK1 (*r* = 0.750; *p* = 0.005), RIPK3 (*r* = 0.684; *p* = 0.014), FADD (*r* = 0.758; *p* = 0.004), and CASP8 (*r* = 0.884; *p* = 0.000) was positively related to ZBP1 levels (Figure 9) in the laser group after *S. globosa* infection, indicating that the ZBP1-PANoptosome pathway was activated after laser irradiation against *S. globosa* infection.

### PANoptosis related pyroptosis (CASP1 and GSDMD), apoptosis (CASP3), and necroptosis (MLKL) protein were activated of laser-irradiated mouse after *Sporothrix globosa* infection

PANoptosis is a highly interconnected cell death process that involves the activation of pattern recognition receptors by pathogenic microorganisms, simultaneously initiating pyroptosis, apoptosis, and necroptosis (Christgen et al., 2020). We further investigated whether PANoptosis-related pyroptosis (CASP1-GSDMD), apoptosis (CASP3), and necroptosis (MLKL) proteins (Bertheloot et al., 2021) were activated in laser-irradiated mice after *S. globosa* infection.

**Figure 10:**
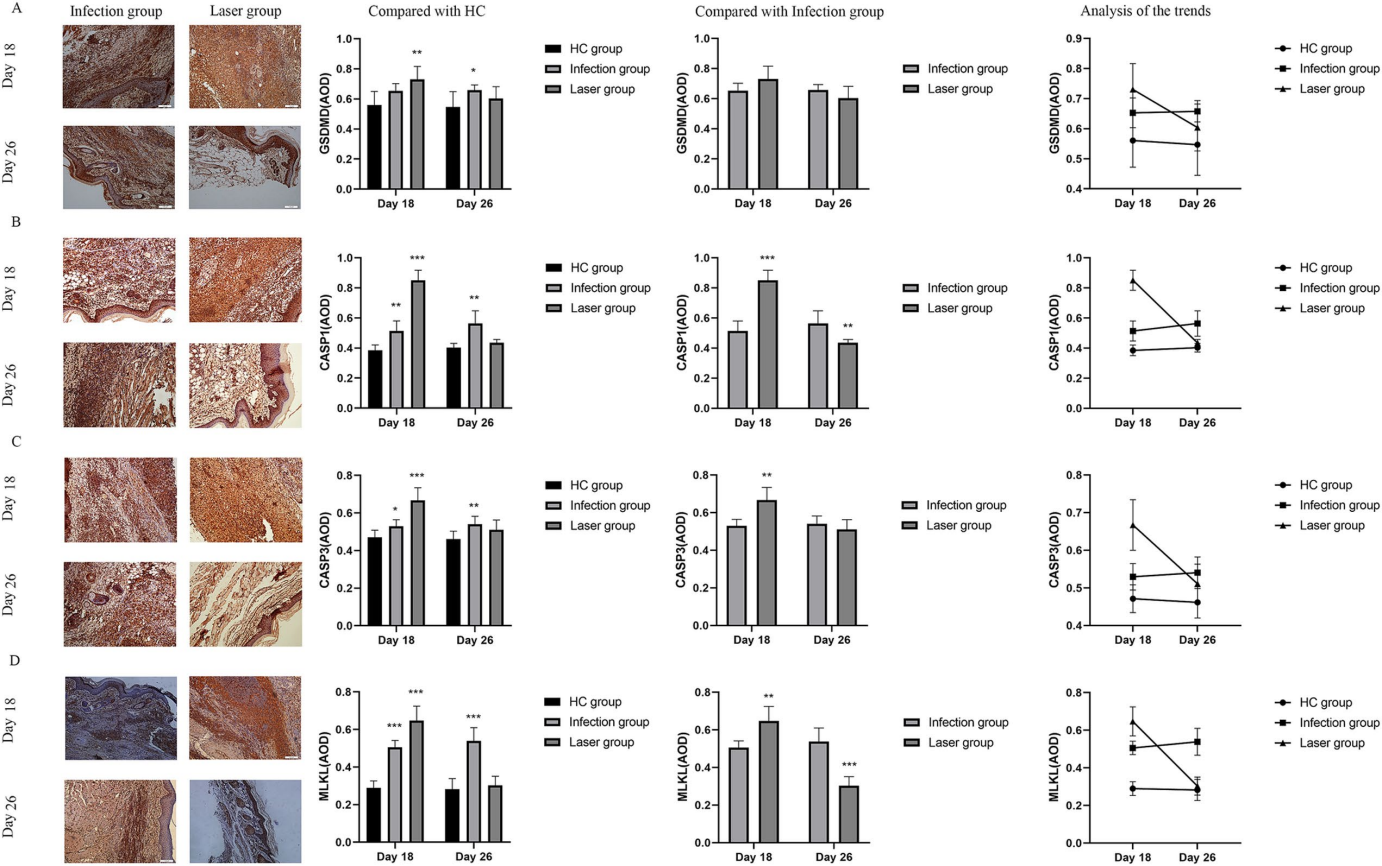
Immunohistochemical analysis of PANoptosis related protein expression levels in the tissues of *S. globosa*-infected mice after different treatments on day 18 and 26. **(A)** GSDMD. **(B)** CASP1. **(C)** CASP3. **(D)** MLKL. Red agent was added to distinguish positive staining from skin pigmentation, with a positive result considered as red staining. Magnification: ×200. GSDMD, gasdermin D; CASP1, caspase-1; CASP3, caspase-3; MLKL, mixed lineage kinase domain-like; AOD, average optical density. ^*^p < 0.05, ^**^p < 0.01, and ^***^p < 0.001 indicate statistical significance.

**Figure 11:**
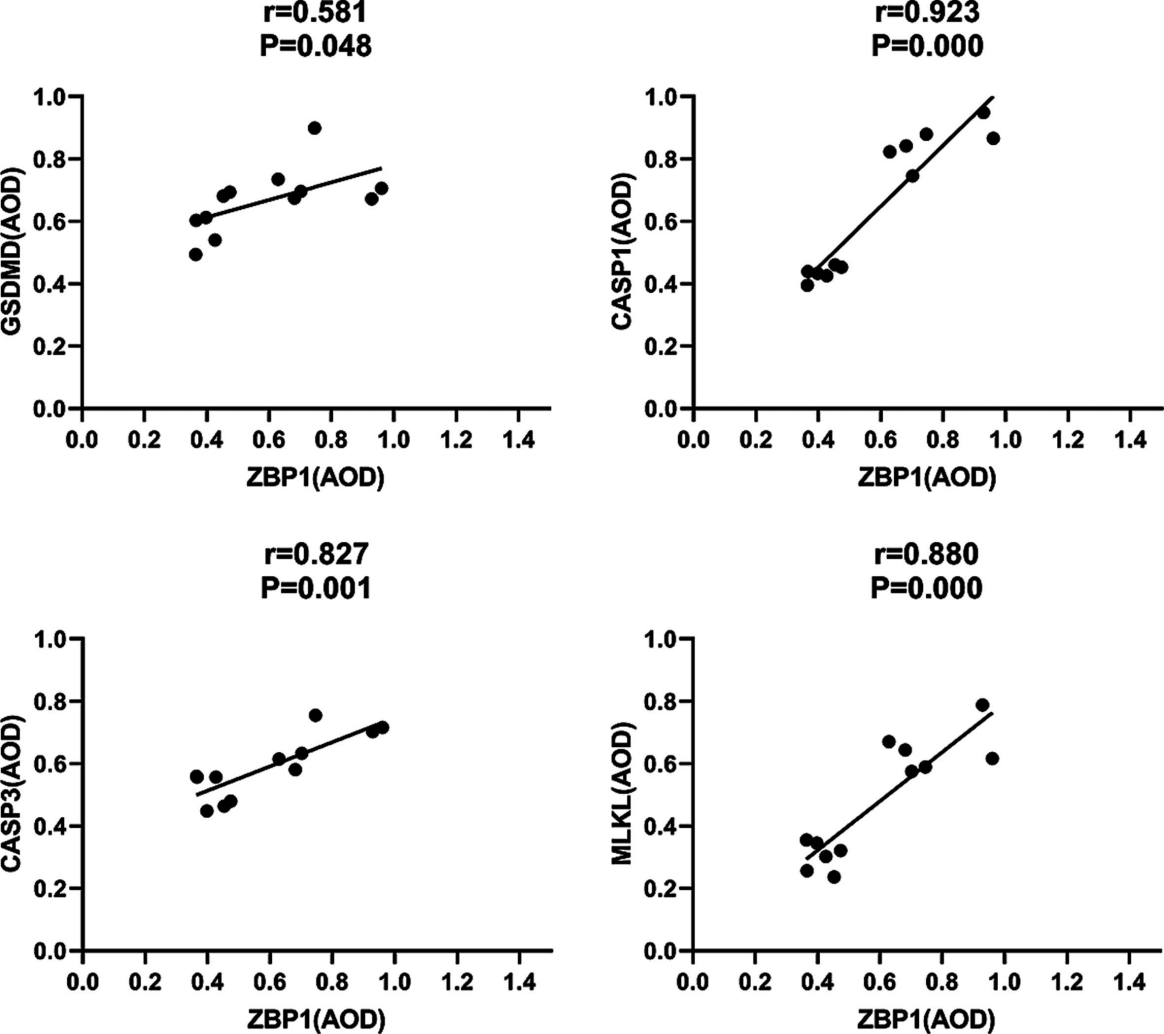
Correlations between ZBP1 and GSDMD, CASP1, CASP3 and MLKL levels in *S. globosa*-infected mice after laser irradiation. Positive correlations between ZBP1 and GSDMD, CASP1, CASP3, MLKL levels after laser irradiation by IHC. AOD, average optical density.

Pyroptosis is induced by the activation of caspase-1 by inflammasomes. Caspase-1 can directly induce cell membrane perforation by cleaving the GSDMD protein, promoting the release of intracellular substances, and causing an inflammatory response (Burdette et al., 2021). In the *in vivo* study, we measured the expression of CASP1 and GSDMD in mouse footpad tissues from different groups. Compared with the HC group, our data showed that CASP1 (0.85 ± 0.07, 0.38 ± 0.04; *p* < 0.001) and GSDMD (0.73 ± 0.09, 0.56 ± 0.09; *p* = 0.007) were all upregulated in the laser group on day 18. Furthermore, the expression of CASP1 in the laser group were significantly higher than that in the infection group (0.51 ± 0.07; *p* < 0.001), indicating that the pyroptosis was activated by laser treatment after *S. globosa* infection *in vivo*. On day 26, the CASP1 (0.56 ± 0.08, 0.40 ± 0.03; *p* = 0.001) and GSDMD (0.66 ± 0.04, 0.55 ± 0.10; *p* = 0.031) levels of the infection group was still higher than that of the HC group, whereas the CASP1 (0.43 ± 0.02; *p* = 0.062) and GSDMD (0.60 ± 0.08; *p* = 0.301) levels returned to almost normal in the laser group (Figures 10A,B). In addition, we found that CASP1 (*r* = 0.923, *p* = 0.000) and GSDMD (*r* = 0.581, *p* = 0.048) were positively correlated with ZBP1 levels (Figure 11) in the laser group after *S. globosa* infection, indicating that PANoptosis was involved in the laser irradiation against *S. globosa* infection by ZBP1-PANoptosome-CASP1-GSDMD pyroptosis pathway.

CASP3 is a critical regulator of apoptosis (Yan et al., 2021b). In the *in vivo* analysis, we measured the expression of caspase-3 in mouse footpad tissues from different groups. Compared with the HC group (0.47 ± 0.04), our data showed that caspase-3 levels were upregulated in the laser (0.67 ± 0.07; *p* < 0.001) and infection (0.53 ± 0.04; *p* = 0.019) groups on day 18. Furthermore, the expression of CASP3 in the laser group was significantly higher than that in the infection group (*p* = 0.001), indicating that apoptosis was activated by laser treatment *in vivo*. On day 26, the CASP3 levels of the infection (0.54 ± 0.04; *p* = 0.008) group was higher than that of the HC (0.46 ± 0.04) group, whereas those of the laser group return to almost normal (0.51 ± 0.05; *p* = 0.100) (Figure 10C). In addition, we found that CASP3 (*r* = 0.827; *p* = 0.001) were positively related to the ZBP1 levels (Figure 11) in the laser group after *S. globosa* infection, indicating that PANoptosis was participating in laser irradiation against *S. globosa* infection via ZBP1 the PANoptosome-CASP3 apoptosis pathway.

Necroptosis is a programmed cell death initiated by RIPK1 and RIPK3 and is executed by the downstream substrate MLKL (Bertheloot et al., 2021). In the *in vivo* study, we measured MLKL expression in mouse footpad tissues from the different groups. Compared with the HC group (0.29 ± 0.04), our data showed that MLKL levels were upregulated in the laser (0.65 ± 0.08; *p* < 0.001) and infection (0.51 ± 0.04; *p* < 0.001) group on day 18. Furthermore, MLKL expression in the laser group was significantly higher than that in the infection group (*p* = 0.002), indicating that necroptosis was activated by laser treatment *in vivo*. On day 26, the MLKL (0.54 ± 0.07; *p* < 0.001) levels of the infection group were higher than that of the HC (0.28 ± 0.06) group, whereas those of the laser group returned to almost normal (0.30 ± 0.05; *p* = 0.515) (Figure 10D). In addition, we found that MLKL (*r* = 0.880; *p* = 0.000) were positively related to the ZBP1 levels (Figure 11) in the laser group after *S. globosa* infection, indicating that PANoptosis was involved in laser irradiation against *S. globosa* infection via ZBP1-PANoptosome-MLKL necroptosis pathway.

### IFN-γ and IL-17 levels of laser-irradiated mouse after *Sporothrix globosa* infection

**Figure 12:**
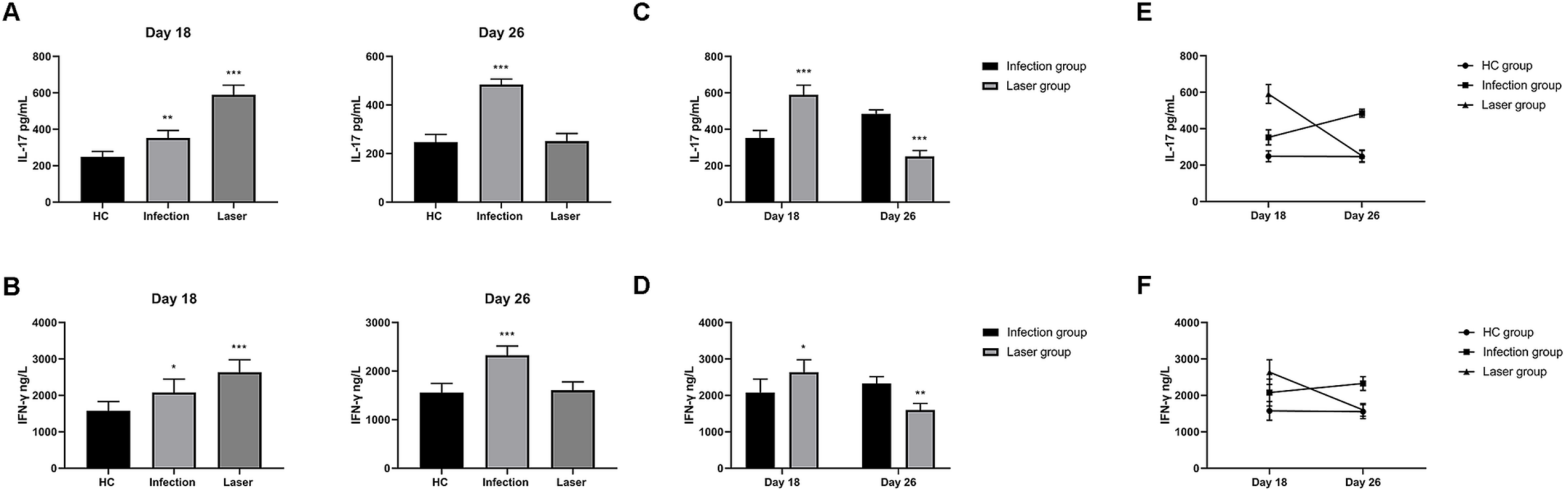
The IL-17 and IFN-γ levels in peripheral blood samples from *S. globosa* infected mice after different treatments. We compared the levels of IL-17 and IFN-γ on day 18 and 26. **(A,B)** The IL-17 and IFN-γ levels in mice from the infection and laser groups compared to the levels in mice from the HC group. **(C,D)** The IL-17 and IFN-γ levels in mice from the laser group compared to the levels in mice from the infection group. **(E,F)** Analysis of the trends of the IL-17 and IFN-γ levels in different groups. Statistical significance regarding the times of treatment was determined. ^*^p < 0.05, ^**^p < 0.01, and ^***^p < 0.001.

IL-1β and IL-18 released by PANoptotic cells act as alarmins initiating and amplifying the inflammatory response (Samir et al., 2020). Previous studies have confirmed that PANoptosis promoted cytokine IL-1β and IL-18 production (Samir et al., 2020), which was related to CD4^+^T cell immunity (van de Veerdonk et al., 2011). IFN-γ and IL-17 are the main cytokines secreted by Th1 and Th17 cells, respectively. We further compared the levels of IFN-γ and IL-17 in peripheral blood on days 18 and 26 after infection (Table 3 and Figure 12).

**Table 3:** Peripheral blood IL-17 and IFN-γ levels assay.

Parameters	Study group	Day 18	Day 26
		(*n* = 6)	(*n* = 6)
	HC group	248.27 ± 29.94	247.01 ± 31.71
IL-17 (pg/mL)	Infection group	352.36 ± 41.64^**^	484.91 ± 21.96^***^
	Laser group	590.57 ± 51.58^***^	250.79 ± 31.91
	HC group	1573.96 ± 257.19	1555.21 ± 190.12
IFN-γ (ng/L)	Infection group	2078.13 ± 369.52^*^	2327.08 ± 188.94^***^
	Laser group	2639.58 ± 338.95^*^	1603.13 ± 176.28

Normally distributed data are shown as the mean ± SD. ^*^Mean compared to HC, *p* < 0.05. ^**^Mean compared to HC, *p* < 0.01. ^***^Mean compared to HC, *p* < 0.001.

Compared to the HC (248.27 ± 29.94 pg/mL) group, we found that the mean IL-17 levels were all upregulated in the laser (590.57 ± 51.58 pg/mL; *p* < 0.001) and infection (352.36 ± 41.64 pg/mL; *p* = 0.001) groups on day 18 (Figure 12A). Furthermore, IL-17 levels in the laser groups were significantly higher than those in the infection group (*p* < 0.001) (Figure 12C), indicating that IL-17 was activated after *S. globosa* infection, and laser treatment further strengthened its expression *in vivo*. In addition, on day 26, we found that the IL-17 levels of the infection group (484.91 ± 21.96 pg/mL; *p* < 0.001) were higher than those of the HC group (247.01 ± 31.71 pg/mL), whereas the levels returned to almost normal in the laser group (250.79 ± 31.91 pg/mL; *p* = 0.841) (Figure 12A). Furthermore, IL-17 levels in the laser group were significantly lower than those in the infection group (*p* < 0.001) (Figure 12C).

Compared to the HC (1573.96 ± 257.19 ng/L) group, we found that the mean IFN-γ levels were all upregulated in the laser (2639.58 ± 338.95 ng/L; *p* = 0.021) and infection (2078.13 ± 369.52 ng/mL; *p* = 0.021) groups on day 18 (Figure 12B). Furthermore, the IFN-γ levels in the laser group were significantly higher than those in the infection group (*p* < 0.001) (Figure 12D), indicating that the IFN-γ levels were activated after *S. globosa* infection, and laser treatment further strengthened its expression *in vivo*. In addition, on day 26, we found that the IFN-γ levels of the infection group (2327.08 ± 188.94 ng/L; *p* < 0.001) were higher than those of the HC group (1555.21 ± 190.12 ng/L); however, the levels returned to almost normal in the laser group (1603.13 ± 176.28 ng/L; *p* = 0.660) (Figure 12B). Furthermore, the IFN-γ levels in the laser group were significantly higher than those in the infection group (*p* = 0.008) (Figure 12D).

These results indicate that laser irradiation may play an antifungal role by regulating PANoptosis-related CD4^+^T cell immune cytokine production (Figures 12E,F).

## Discussion

In this study, we used IHC to analyze the ZBP1 levels in patients with sporotrichosis. Higher levels of ZBP1 were observed during the early phases of the disease. This suggests that ZBP1 may be reactive in the initial stages, which helps the body to resist *S. globosa* infections. Besides, our *in vitro* results showed that laser irradiation (at an energy density of 114 J/cm^2^) was highly effective, inducing morphological changes, Hoechst/PI apoptosis and necroptosis, DNA fragmentation, calcium determination, and metacaspase activation in *S. globosa*. These results indicate that laser treatment probably induces *S. globosa* apoptosis and necroptosis, accompanied by significant changes in nucleic acid levels. ZBP1 is a key innate immune sensor for endogenous nucleic acid ligands that can detect nucleic acid damage and release, leading to PANoptosis. Moreover, our *in vivo* results showed that skin lesions and histopathological changes resolved after laser treatment. In addition, we found that the levels of ZBP1, PANoptosomes (RIPK1, RIPK3, FADD, CASP8), PANoptosis-related pyroptosis (CASP1, GSDMD), apoptosis (CASP3), and necroptosis (MLKL) proteins were upregulated simultaneously in the footpads of mice that received laser treatment, showing a positive relationship. Elevated expression of the main cytokines IL-17 and IFN-γ secreted by Th1 and Th17 cells after laser treatment. These results suggest that the Nd:YAGl064nm laser caused changes in the nucleic acid levels in *S. globosa*. Immune response-related macrophages perceive changes in nucleic acid levels to recognize *S. globosa* and induce PANoptosis by activating the PANoptosome complex to combat sporotrichosis.

PANoptosis is a newly discovered, highly interconnected programmed cell death pathway in which pathogenic microorganism infection activates the pattern recognition receptor and concurrently engages in pyroptosis, apoptosis, and necroptosis (Christgen et al., 2020). Z-DNA binding protein 1, abbreviated as ZBP1, is a nucleic acid sensing protein that includes Zα2 domain for binding to Z-DNA and Z-RNA and RHIM domain for conducting signals (Jiao et al., 2022). Recent studies have shown that ZBP1, as a central regulator of PANoptosis, can regulate cell apoptosis, pyroptosis, and programmed necrosis through novel regulatory necrosomes (PANoptosomes), thereby helping the host resist infection (Samir et al., 2020; Zheng and Kanneganti, 2020; Malireddi et al., 2023). Previous study found that ZBP1 is a key innate immune sensor of endogenous nucleic acid ligands (Zheng and Kanneganti, 2020). The Zα2 domain of ZBP1 sensed changes of the DNA and RNA levels in influenza A virus (IAV)-infected cells, activating the conserved RHIM domain to interact with RIPK1/RIPK3 and induced PANoptosis. In the presence of RIPK3, ZBP1 induces cell apoptosis via the RIPK1-RIPK3-Caspase-8 axis, and MLKL-mediated necroptosis, and activation of the NLRP3 inflammasome promotes pyroptosis. However, in the absence of RIPK3, ZBP1 binds only to RIPK1 and causes apoptosis, whereas ZBP1 deficient cells are less likely to undergo programmed cell death after IAV (Kesavardhana et al., 2020; Kuriakose et al., 2016). Banoth et al. (2020) demonstrated that PANoptosis-related apoptosis (CASP3/7/8), pyroptosis (CASP1 and GSDMD), and necroptosis (MLKL) proteins were significantly upregulated in macrophages infected with *C. albicans* or *A. fumigatus*. Moreover, loss of PANoptosomes (RIPK1, RIPK3, CASP8, and FADD) inhibits macrophage death induced by *C. albicans* or *A. fumigatus* infection. The macrophages of ZBP1^−/−^ or ZBP1^ΔZα2/ΔZα^ mice are less susceptible to infection with *C. albicans* and *A. fumigatus*, indicating that both agents can pass through the Zα2 domain of ZBP1 and promote the formation of PANoptosome (RIPK1, RIPK3, CASP8, and FADD) complexes, thereby activating PANoptosis by pyroptosis (NLRP3, ASC, CASP1/11, GSDMD), apoptosis (CASP3/7/8), and necroptosis (RIPK3/RIPK1, pMLKL). However, no study has shown whether ZBP1 participates in anti-sporotrichosis effects. In this study, we found that the expression of ZBP1 in the skin lesions of patients with sporotrichosis was significantly higher than that in normal skin tissues (*p* < 0.001). The expression of ZBP1 in sporotrichosis tissues was not related to the patients sex or age (*p* > 0.05) but was related to the duration of the disease. The expression level of ZBP1 in skin lesions in the acute group was significantly higher than that in the non-acute group (*p* < 0.001). These results indicate that high ZBP1 expression may play an important role in the occurrence and development of sporotrichosis and is expected to become a novel target for the diagnosis and treatment of sporotrichosis.

Sporotrichosis affects humans and domestic animals. In recent years, the global incidence rate of sporotrichosis has increased, and searching for effective targets and treatment methods related to this disease has important theoretical significance and practical clinical application value for the diagnosis, evaluation, and treatment of sporotrichosis (Xavier et al., 2023; Uemura and Rossato, 2023). With the continuous deepening of research, the effect of laser on nucleic acids has been confirmed. Li et al. (2017) found that Nd:YAGl064nm laser irradiation can induce heritable DNA methylation. Barrera et al. (2021) proposed that ZBP1 is a pattern recognition receptor for mitochondrial components. Reactive oxygen species accumulation can attack mitochondria, inhibit mitochondrial respiratory function, reduce mitochondrial membrane potential, and promote the release of mitochondrial injury-related molecular patterns such as cardiolipin, mitochondrial DNA (mtDNA), and mitochondrial formylation peptides, thereby activating the ZBP1 protein and inducing an inflammatory response. Our recent study showed that a Nd:YAGl064nm laser can selectively damage the cell wall of *S. globosa*, blocking the cell cycle in the S phase and affecting DNA synthesis (Yan et al., 2021a). In this study, we also confirmed that laser irradiation could alter nucleic acids through morphological and structural changes, DNA fragmentation, and nuclear condensation, which are key triggers for ZBP1 activation.

In this study, we showed that the PANoptosis-related ZBP1-FADD-CASP8-CASP3 apoptotic pathway was activated in laser-irradiated mice after *S. globosa* infection. SEM confirmed the shrinkage of the *S. globosa* surface and leakage of the content after laser treatment. Moreover, Hoechst/propidium iodide double staining further indicated that laser treatment maybe caused apoptosis of *S. globosa*. Apoptosis is characterized by mitochondrial membrane potential shifts, mitochondrial reactive oxygen species levels, and the apoptotic protein metacaspase (Sakao et al., 2023; Zaib et al., 2022), which was confirmed in our previous study (Yan et al., 2021a). In addition, we found that cytoplasmic Ca^2+^ levels increased after laser treatment, which is an important apoptosis-inducing signal that causes conformational changes in the ANT and open PT pores. The continuous opening of PT pores promotes a series of changes in the mitochondria, including uncoupling of the respiratory chain, increased mitochondrial matrix osmotic pressure, and inner membrane swelling. This is accompanied by the release of mitochondrial proteins, such as apoptosis-inducing factor, cytochrome C, and endonuclease G, ultimately leading to cell apoptosis (Fujikawa, 2015; Liu et al., 2023). Moreover, ZBP1, FADD, CASP8, and CASP3 are critical mediator of apoptosis, research has shown that the Fas/FasL-FADD-CASP8-CASP3/7 cell apoptosis pathway in the death receptor pathway is closely related to fungal PANoptosis (Banoth et al., 2020). Fas binds to FasL to form an activated FasL trimer, and its intracellular DD interacts with the DD at the C-terminus of the FADD. FADD binds to the caspase-8 precursor through its N-terminal death effector domain to form a death-inducing signaling complex (DISC). After DISC formation, it can induce the self-activation of CASP8, and the activated CASP8 further activates the downstream CASP3/7 cascade reaction, leading to cell apoptosis (Wang and Su, 2018). The liver is an important barrier for controlling blood transmission of *C. albicans*. Renna et al. (2015) found that *C. albicans* infection can promote liver injury, including apoptosis, by upregulating FasL expression in liver NK and NKT cells. In this study, we investigated the expression of ZBP1, FADD, CASP8, and CASP3 *in vivo* using IHC. The results showed that they were all upregulated by the laser treatment. FADD, CASP8, and CASP3 positively correlated with ZBP1 levels.

In this study, we confirmed that the PANoptosis related ZBP1-NLRP3-CASP1-GSDMD pyroptosis pathway was activated in laser-irradiated mice after *S. globosa* infection. The classic pathway of pyroptosis is induced by the activation of CASP1 by inflammasomes. Inflammasomes are protein complexes composed of receptor proteins such as NLRP1, NLRP3, AIM2, apoptosis-related speckled protein (ASC), and the effector molecule CASP1. Danger signals, such as pathogen infection and tissue damage, are recognized and bound to cell receptor proteins through relevant molecular patterns, triggering ASC recruitment of procascase-1 assembly to form inflammasomes and promoting its lysis to form CASP1. CASP1 can directly induce cell membrane perforation by cleaving GSDMD, promoting intracellular substance release, and inducing an inflammatory response (Burdette et al., 2021). Besides, CASP1 can induce cytokine IL-1β and IL-18 production, leading to inflammation and promoting pyroptosis (Zhen and Zhang, 2019; Zhao and Zhao, 2020). IL-1β promotes the differentiation of Th17 cells and the aggregation of eosinophils, while maintaining the production of Th17 related cytokines. IL-18 enhances the Th1 cell-mediated immune response and promotes the secretion of mast cells and basophils. NLRP3 inflammasome-induced cell pyroptosis is thought to be associated with CD4^+^T cell immunity (Yan et al., 2021b). Gonçalves et al. (2017) found that NLRP3, ASC, and CASP1 knockout mice were more susceptible to sporotrichosis, and the levels of Th1 and Th1/Th17 cells in the knockout mice were significantly reduced, indicating that NLRP3 inflammasomes have an anti sporotrichosis effect, which is related to cell pyroptosis and CD4^+^ T cell immunity. In this study, we confirmed through IHC that ZBP1, NLRP3, CASP1, and GSDMD are critical mediators of pyroptosis and explored their expression changes *in vivo* using IHC. The results showed that they were all upregulated by laser treatment and that NLRP3, CASP1, and GSDMD were positively correlated with ZBP1 levels. Besides, we found that the levels of IFN-γ and IL-17 in *S. globosa*-infected mice changed significantly after laser treatment, whereas the levels recovered faster than what was seen in the untreated infected mice.

In this study, we confirmed that the PANoptosis-related ZBP1-RIPK3/RIPK1-MLKL necroptosis pathway was activated in laser-irradiated mice after *S. globosa* infection and that programmed cell death was a regulated mode of programmed cell death initiated by receptor-binding serine/threonine protein kinases 1 and 3 (RIPK1 and RIPK3), executed by downstream substrate mixed lineage kinase domain-like proteins (MLKL), controlled by their unique signaling pathways. RIPK1 and RIPK3 are important regulatory proteins in the programmed necrosis signaling pathway (Vandenabeele et al., 2010). RIPK1 is a key determinant of cell death. Its main biological function is to induce cell apoptosis and programmed necrosis, and it plays a crucial role in the TNF-kB signaling pathway. RIPK3 is the key to inducing the conversion between cell apoptosis and necrosis. Programmed cell necrosis occurs via both classical and non-classical pathways. The non-classical pathway of necrosis can be mediated by the stimulation of pathogenic microorganisms with lipopolysaccharides, which causes RIPK3 phosphorylation through the ZBP1 receptor, leading to programmed necrosis. Zhang et al. (2020) reported the “inside out” pathway of cell death initiated by ZBP1 from the nucleus to the plasma membrane after infection with influenza A virus. ZBP1 induces influenza A virus RNA and activates RIPK3 to initiate RIPK3 driven cell death. Replication of the influenza A virus produces Z-RNA, activating ZBP1 in the infected cell nucleus. ZBP1 subsequently initiates the RIPK3 mediated MLKL pathway in the nucleus, leading to nuclear membrane rupture, cytoplasmic DNA leakage, and necrosis. Nuclear MLKL-induced cell death is an effective activator of neutrophils, promoting their ability to drive inflammatory pathology in highly toxic influenza viral diseases. Therefore, MLKL-deficient mice exhibit reduced destruction of lung epithelial cell nuclei, reduced recruitment of neutrophils to infected lungs, and increased survival rate after a lethal dose of influenza virus. In this study, we demonstrated that the PANoptosis-related necroptosis pathway is activated in laser-irradiated *S. globosa*. After laser treatment, SEM experiment showed significant shrinkage of the *S. globosa* cell surface and a large amount of leakage of cell contents. Moreover, using Hoechst/propidium iodide double staining, we further found that laser treatment probably caused necroptosis of *S. globosa*. Thus, we speculate that necroptosis is a major mechanism of laser therapy against *S. globosa*. Moreover, ZBP1, RIPK3, RIPK1, and MLKL are critical mediators of necroptosis, and we explored changes in their expression *in vivo* using IHC. The results showed that they were all upregulated by laser treatment and that RIPK3, RIPK1, and MLKL were positively correlated with ZBP1 levels.

Our study has several limitations. The ZBP1 inhibitor ADAR1 weakened the antifungal effect of the Nd:YAG1064nm laser, which needs to be studied in the future. A larger sample size would improve the reliability of the statistical analysis. Further studies with larger samples are needed, and the long-term effects of laser treatment need to be further investigated.

## Conclusion

The results of this study demonstrate the key PANoptosis regulatory protein, ZBP1, was high expression in the sporotrichosis. Moreover, Nd:YAG1064nm laser effectively inhibited the growth of *S. globosa* by activating fungal PANoptosis. During this process, the morphological structure, Hoechst/PI apoptosis and necroptosis analysis, DNA fragmentation, calcium determination, and metacaspase activation of *S. globosa* changed significantly. In addition, we confirmed that the Nd:YAG1064nm laser activated the ZBP1-PANoptosome-PANoptosis pathway by identifying changes in nucleic acid levels and by regulating the immunity of Th1 and Th17 cells. In conclusion, our results provide a theoretical basis for the clinical application of an Nd:YAG1064nm laser for the treatment of sporotrichosis.

## Data Availability

The original contributions presented in the study are included in the article/supplementary material, further inquiries can be directed to the corresponding authors.
